# Compositional and functional profiling of the rhizosphere microbiomes of the invasive weed *Ageratina adenophora* and native plants

**DOI:** 10.7717/peerj.10844

**Published:** 2021-03-04

**Authors:** Yun Xia, Minghua Dong, Lei Yu, Lingdong Kong, Robert Seviour, Yunhong Kong

**Affiliations:** 1 School of Agriculture and Biotechnology, Kunming University, Kunming, Yunnan, China; 2 Microbiology Department, La Trobe University, Bundoora, Victoria, Australia; 3 Kunming Key laboratory of Hydro-Ecology Restoration of Dianchi Lake, Kunming University, Kunming, Yunnan, China

**Keywords:** Plant-soil feedback, Exotic invasion, High throughput 16S rRNA and ITS fragment sequencing, Transcriptome analysis, Rhizosphere microbiome, *Ageratina adenophora*

## Abstract

The rhizosphere soil microbiome (RSM) plays an important role in the nutritional metabolism of the exotic weed *Ageratina adenophora*. However, our understanding of the composition and metabolic activity of this microbiome is limited. We used high-throughput sequencing of bacterial 16S rRNA genes and fungal internal transcribed spacer fragments in combination with transcriptome analysis to compare the composition and metabolic features of the RSMs of *A. adenophora* and the native plant species *Artemisia indica* and *Imperata cylindrica*. *A. indica* cohabitates with the weed and *I. cylindrica* grows in uninvaded soil areas. We found fungi belonging to the phyla Ascomycota and Basidiomycota and bacteria belonging to the phyla Proteobacteria, Acidobacteria and Bacteroidetes were highly abundant in the RSMs of *A. adenophora* and both native plant species. The RSM of *A. adenophora* differed to varying degrees in the relative abundances of bacterial and fungal phyla and genera, and in levels of expression of functional genes from those of both the native species. The RSM of *A. adenophora* was more metabolically active than both of these, as indicated by marked increases in the expression levels of genes associated with cell wall, membrane, and envelope biogenesis, energy production and conversion, and the transport and metabolism of carbohydrates, amino acids, coenzymes, nucleotides, and secondary metabolites. Ascomycota and Basidiomycota contributed most significantly to these differences. The composition and metabolic activities of *A. adenophora* RSM differed less to the RSM of *A. indica* than to the RSM of *I. cylindrica*. Fungal communities contributed most to the metabolic genes in the RSM of *A. adenophora.* These included the arbuscular mycorrhizal fungi Glomeromycota. The different relative abundances in the RSMs of these three plant populations may explain why *A. adenophora* is more successful in colonizing soils than the two native populations.

## Introduction

*Ageratina adenophora* (Sprengel, also known as *Eupatorium adenophorum* Sprengel) is a weed originating in Mexico and Costa Rica that has invaded more than 40 tropical and subtropical countries in Asia, Oceania, Africa*,* and Europe *(Poudel et al., 2019*). Its dominance can interfere with nutritional cycles, hydrological conditions, and the energy budgets of the plant-soil ecosystem, causing severe economic losses to the agricultural, forestry, and livestock industries (*Xu et al., 2006*; Kong et al., 2017). Thus, *A. adenophora* is often used as a model organism to study the possible mechanisms of plant invasion (*Datta et al., 2017*; *Chen et al., 2019*; *Fang et al., 2019; Poudel et al., 2019*; *Zhao et al., 2019*). The impact of plant soil feedback is of interest to explain the successful exotic invasion of *A. adenophora* (*Niu et al., 2007; Poudel et al., 2019; Zhao et al., 2019*). The presence of *A. adenophora* may alter soil nutrient availability by modifying the composition and function of its soil microbiome, thus facilitating its growth and competitiveness (*Niu et al., 2007; Zhao et al., 2019*).

Most soil microbiological studies associated with the invasion of *A. adenophora* have examined the bulk soil beneath the plant. *A. adenophora* tends to have higher levels of available C, N, and P (*Niu et al., 2007; Kong et al., 2017*; *Poudel et al., 2019*; *Zhao et al., 2019*), higher nitrogen-cycling rates (*Zhao et al., 2019*), higher enzymatic (urase, phosphatase, and invertase) activities (*Li et al., 2009*), and a higher abundance of vesicular-arbuscular mycorrhizal fungi and fungi/bacteria ratio (*Niu et al., 2007*) than soils inhabited by native plant species. In previous studies, the composition of the bulk soil microbiomes beneath *A. adenophora* differed from those beneath native plant species (*Niu et al., 2007; Kong et al., 2017; Zhao et al., 2019*). However, there have been few studies conducted on the structure and function of the rhizosphere soil microbiome (RSM) of this weed. The soil immediately around the plant roots, known as the rhizosphere, is an area where plant soil feedback is important because it harbors an active and diverse microbiota consisting of both bacteria and fungi (Inderjit & Van der Putten, 2010; Van der Putten et al., 2013). These microorganisms receive 20%–50% of the plant host’s photosynthetically generated carbon (*Drigo et al., 2010*). However, the extent to which the composition and metabolism of the bacterial and fungal communities in the RSM of *A. adenophora* differ from those of native plant species is still unclear. In particular, the root leachate of *A. adenophora* contains structurally diverse phytochemicals including terpenoids, phenylpropanoids, flavonoids, coumarins, and alkaloids (*He et al., 2008; Dong et al., 2017*), and some of these have been shown to possess allelopathic, phytotoxic, and antifeedant activities, and have the potential to substantially alter the microbiota of invaded soils (*Liu et al., 2010*).

We investigated the composition and metabolic activities of the bacterial and fungal communities in the RSMs of *A. adenophora* and compared them to those of two native plant species, *Artemisia indica* and *Imperata cylindrica,* using 16S rRNA gene and internal transcribed spacer (ITS) fragment high-throughput sequencing combined with transcriptome analysis. The results of this study improve our understanding of the role of RSM in the growth and invasiveness of *A. adenophora*.

## Materials & Methods

### Site description and sampling strategy

**Figure 1:**
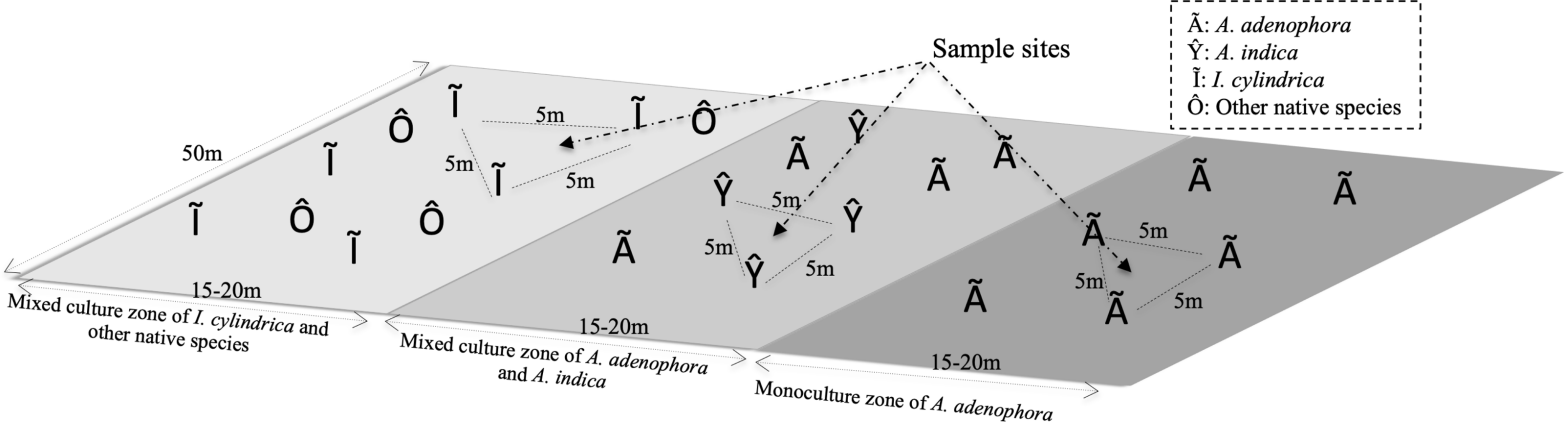
Diagram of the rhizosphere soil collection sites of weed *Ageratina adenophora*, and native species *Artemisia indica* and *Imperata cylindrica.*

The study site was located in the foothills of Maomao Qing, Xishan, Kunming (24°55′29″N, 102°36′36″E), where the invasion of *A. adenophora* began in 1987. The vegetation of the study area consisted of a monoculture of *A. adenophora,* a mixture of *A. adenophora* (40%–50% coverage) and the native species *A. indica*, and *I. cylindrica* (Fig. 1). *I. cylindrica* was initially present in the sample site area but was outcompeted by *A. adenophora* and disappeared. The sample site was approximately 50 × 50 m^2^ with a mean altitude of 2,170 (±0.5) m above sea level and with no tree coverage. The site had mountain red soil, an average annual temperature of 15.2 °C (ranging from 14.3–16.1 °C), and an average annual rain precipitation of 975 mm ranging from 937–1,058 mm.

To identify the rhizosphere microbial populations and to determine their roles in the metabolic activities of the RSM of *A. adenophora*, triplicate soil samples were collected from the rhizospheres of *A. adenophora* in the monoculture zone, *A. indica* in the mixed zone, and *I. cylindrica* where no invasion of *A. adenophora* had occurred (Fig. 1). Nine total rhizosphere samples were obtained. The individual sample sites in each zone were separated by at least 5 m. Sample sites in the different zones were separated by at least 10 m. To sample the rhizosphere soils of each species, individual plants were pulled up within an area of approximately 80 × 80 cm^2^ and shaken to remove the soil loosely attached to the roots, ensuring only soil firmly adhering to the roots was sampled. A subset of these soil samples was frozen in liquid nitrogen for RNA-seq analysis. Another set was placed separately into sterile Whirl-Pak sample bags (Nasco, Fort Atkinson, WI, USA) and transported to the laboratory within 1 h of collection where it was used for 16S rRNA gene and ITS fragment analyses. Approximately 500 g of each soil sample was homogenized (medium treatment for 1 min) in a Waring heavy-duty blender (Lab-Biogen, Kunming, China), which was carefully cleaned with 70% ethanol after each use, and immediately passed through a two mm sieve yielding samples used for subsequent physicochemical analysis and DNA extraction, as detailed below.

### Physicochemical analysis of soil samples

Physiochemical analyses of soil samples were carried out according to the protocols described by Kong et al. (2017), with modifications. Briefly, soil mass water content was determined by measuring the percent weight loss after 24 h incubation at 105 °C. Soil organic matter composition was determined using the K_2_Cr_2_O_7_-H_2_SO_4_ oxidation method (*Nelson & Sommers, 1996*). Total nitrogen was measured using the Kjeldahl method. Soil inorganic N was extracted with 2 mol KCl, and the concentrations of NO_3_^−^-N and NH_4_^+^-N in the KCl extracts were determined using hydrazine sulfate colorimetry and indophenol blue colorimetry, respectively (*Mulvaney, 1996*). The total and available phosphorus levels were determined using the colorimetric Moblue-method (*Kuo, 1996*), while the total and available potassium levels were measured using the method described by Helmke & Sparks (1996). The pH values (1:2.5 solution of soil to water) were measured using a pH meter (Mettler-Toledo International Inc., Columbus, OH, USA).

### DNA extraction, PCR amplification, and Illumina sequencing

DNA extraction, PCR amplification, and Illumina sequencing were carried out according to the protocols described by Kong et al. (2017) and Xia et al. (2019a) with modifications. Briefly, soil DNA was extracted using a PowerSoil DNA Isolation Kit (Anbiosci, Shenzhen, China) according to the manufacturer’s protocol. Crude DNA was purified on 1% (wt/vol) low melting point agarose gel in Tris Acetate-EDTA buffer to obtain genomic DNA. DNA bands were excised, extracted with QIAquick Gel Extraction Kits (Qiagen, Shanghai, China), and then used as templates for PCR amplification. The V4 hypervariable region of the 16S rRNA gene *(Biddle et al., 2008*) was amplified using 2 × KAPA HiFi HotStart ReadyMix (Shanghai Dobio, Shanghai, China) with the primer sets 515F (GTGCCAGCMGCCGCGGTAA) (*Turner, Pryer & Miao, 1999*) and 907R (CCGTCAATTCMTTTRAGTTT) (Lane, 1991) under the following PCR conditions: initial denaturation at 94 °C for 3 min, followed by five cycles of denaturation at 94 °C for 10 s, annealing at 55 °C for 15 s, and extension at 72 °C for 30 s before a final extension at 72 °C for 5 min. After cleanup with AMPure XP beads, the 16S rRNA gene amplicons were attached to dual indices and Illumina sequencing adapters using a Nextera XT Index Kit (Illumina, Inc., San Diego, CA, USA) under the following PCR (also prepared with 2 × KAPA HiFi HotStart ReadyMix) conditions: an initial denaturation at 95 °C for 30 s followed by eight cycles of denaturing at 95 °C for 30 s, annealing at 55 °C for 30 s, and extension at 72 °C for 30 s before a final extension at 72 °C for 5 min. The PCR amplifications were carried out in triplicate for each sample, and the PCR products were again cleaned with AMPure XP beads, quantified using an Agilent Technologies 2100 Bioanalyzer (Agilent Technologies Inc., Santa Clara, CA, USA), normalized, and pooled. These 16S rRNA gene amplicons were then diluted to 4 nM with 10 mM Tris (pH 8.5) before being denatured with NaOH and sequenced (with 5% PhiX as internal control) on a MiSeq IIlumina Platform (PE250) at Genergy Biotechnology (Shanghai, China).

The ITS2 regions of fungal rRNA were amplified using 2 × KAPA HiFi HotStart ReadyMix (Shanghai Dobio) and the primers ITS3_KYO2 (GATGAAGAACGYAGYRAA) and ITS4_KYO3 (TCCTCCGCTTATTGATATGC) (*Toju et al., 2012*). The PCR conditions were as follows: an initial denaturation of 15 min at 95 °C, followed by 30 cycles of 30 s at 95 °C, 30 s at either 51 °C or 55 °C, and 30 s at 72 °C, and a final elongation for 5 min at 72 °C. The ITS amplicons obtained were then treated and sequenced following the same procedures described earlier for the 16S rRNA gene amplicons.

### Phylogenetic analyses

Phylogenetic analyses were carried out according to the methods described by Kong et al. (2017) and Xia et al. (2019a) with modifications. The V4 amplicons of the 16S rRNA genes and ITS fragments were pair-end assembled and checked using Flash software (Magoc & Salzberg, 2011) to ensure that their sequences matched perfectly with the index sequences, had no more than one mismatch error present in the forward primer sequences, and the trimmed sequences were longer than 200 bp. Then, QIIME (Caporaso et al., 2010) was used to analyze the 16S rRNA and ITS amplicons to generate OTU clusters and perform alpha diversity analyses. The V4 and ITS amplicon sequences were grouped into OTUs at the 97% identity threshold (3% dissimilarity levels) using the RDP classifier (Release 11.1 http://rdp.cme.msu.edu/) and Unite (Release 6.0 https://unite.ut.ee/index.php), respectively. Any OTU represented by ≤3 sequences was removed. The 16S rRNA and ITS amplicon sequences were deposited in the NCBI Sequence Read Archive under Submission ID SUB7457084 and BioProject ID PRJNA633422. Biodiversity indices, including the Chao1 index, Shannon index, and coverage ratios, were calculated with Mothur *(Schloss et al., 2009*) following the procedures provided and again applying a 97% identity threshold.

### RNA extraction, library preparation, and sequencing

RNA extraction, library preparation, and sequencing were conducted according to the methods described by Xia et al. (2019b) with modifications. Briefly, total RNA was isolated using TRIzol reagent (Thermo Fisher Scientific, Waltham, MA, USA) after grinding the frozen soil samples to a fine powder in liquid nitrogen. Equimolar amounts of RNA extracted from three rhizosphere soil samples from each plant were pooled for transcriptomic analysis to determine the active microbial groups in the RSM and to screen the differentially expressed genes in the RSMs of the different plant species. The quality and quantity of the extracted RNAs were monitored using 1% agarose gel before rRNAs were removed using Ribo-Zero rRNA Removal Kits (Qiagen) following the manufacturer’s instructions. For each sample, a library of approximately 200 bp insert sizes was prepared with a TruSeq RNA Sample Prep Kit (Qiagen), and mRNAs were amplified with a bridge PCR using a HiSeq 3000/4000 Cluster Kit (Illumina, Inc.) according to the manufacturer’s instructions. The cDNAs obtained were subjected to 2 × 100 bp paired-end (PE100) sequencing on a HiSeq 2000 instrument using HiSeq 3000/4000 SBS Kits (Illumina, Inc.) on the HiSeq platform of Majorbio Bio-Pharm Technology Co., Ltd., Shanghai, China. The raw RNA sequence dataset supporting the conclusions of this article is available in the NCBI Short Reads Archive (SRA) under the submission ID PRJNA638694.

### Sequence quality control and genome assembly

Sequence quality control and genome assembly were carried out according the protocols described in Xia et al. (2019b). Briefly, we truncated cDNA sequences at the 3′ and 5′ ends using SeqPrep (https://github.com/jstjohn/SeqPrep) and removed adapter contamination (at least 10 Ns from the raw data (FASTQ format)) using Sickle (https://github.com/najoshi/sickle). Then, we assembled them using SOAPdenovo (https://sourceforge.net/projects/soapfuse/, Version 1.06). Scaffolds over 500 bp were extracted and broken into contigs without gaps. Contigs were used for further gene prediction and annotation.

### Gene prediction, taxonomy, and functional annotation

Gene prediction, taxonomy, and functional annotation were carried out according to the protocols described by Wang et al. (2019). Briefly, open reading frames (ORFs) from each soil sample were predicted using MetaGene (http://metagene.nig.ac.jp/metagene/download_mga.html). The predicted ORFs with lengths of more than 100 bp were retrieved and translated to amino acid sequences using the NCBI translation table (http://www.ncbi.nlm.nih.gov/Taxonomy/taxonomyhome.html/index.cgi?chapter=tgencodes#SG1). All sequences from gene sets with a 95% sequence identity (90% coverage) were clustered as a non-redundant gene catalog by CD-HIT (http://www.bioinformatics.org/cd-hit/). After quality control, reads were mapped to the representative genes with 95% identity using SOAPaligner (https://sourceforge.net/projects/soapfuse/), and gene abundances in each sample were evaluated. BLASTP (Version 2.2.28+, http://blast.ncbi.nlm.nih.gov/Blast.cgi) was employed for taxonomic annotations by aligning non-redundant gene catalogs against the NCBI NR database with an e-value cutoff of 1 e^−5^. Clustering of orthologous groups (COG) of proteins for ORF annotation was performed using BLASTP against the eggNOG database (v4.5) with an e-value cutoff of 1 e^−5^. Unigene expression heatmaps in the different rhizosphere soil samples were constructed using R package software (https://www.r-project.org).

### Nitrogen metabolic enzyme annotations

The non-redundant genes obtained from each individual rhizosphere soil sample were matched with KEGG (Kyoto Encyclopedia of Genes and Genomes, http://www.genome.jp/kegg/) and orthologies related to nitrogen metabolism (metabolic pathway ko00910), including ammonification, nitrification, and denitrification, were retrieved from the KEGG database using BLASTP (BLAST Version 2.2.31+, http://blast.ncbi.nlm.nih.gov/Blast.cgi) with an e-value threshold of 1 e^−5^. The number of genes matching individual KEGG orthologies was enumerated.

### Statistical analyses

Average values and relative abundances of bacterial and fungal phyla and genera were calculated using Excel (Version 2010). Significant differences in soil physiochemical parameters between the different plant species were determined using the Student’s *t*-test in Excel. Significant differences in the relative abundance of bacterial and fungal phyla and genera between the RSM of *A. adenophora* and either of the two native plant species were determined using the Kruskal–Wallis test with SPSS (version 16.0). Significant differences were established at *P* ≤ 0.05. Venn figures and gene expression profiles were calculated and drawn using R software (https://www.r-project.org).

## Results

### Physiochemical properties of rhizosphere soils

The physiochemical characteristics of the rhizosphere soil supporting the growth of *A. adenophora* and the two native species are listed in Table 1. The soil parameters supporting only *A. adenophora* differed from those in which the two native species were growing. Thus, levels of total organic matter, total nitrogen and P, available nitrogen and P, and NO_3_^−^-N detected in the rhizosphere soil samples with *A. adenophora* were significantly (*P* < 0.05) higher than those detected in the rhizosphere soil samples from both *A. indica* and *I. cylindrica*. However, total K levels detected in the rhizosphere soil samples from *A. adenophora* were significantly (*P* < 0.05) lower than in rhizosphere soil samples of *A. indica* and *I. cylindrica*. Values for soil pH, total P, available K, and NH_4_^+^-N did not differ significantly among all three rhizosphere soils. The moisture content of the soil samples from *A. adenophora* was higher (*P* < 0.05) than that detected in the rhizosphere soil samples of *A. indica* but was the same as that for *I. cylindrica*.

**Table 1:** Physiochemical characterization of rhizosphere soils of *Ageratina adenophora*, and native plant species *Artemisia indica* and *Imperata cylindrica*.

Rhizospheric soil of plant species	Moisture %	pH	Organic C (g Kg^−1^)	NH_4_^+^-N (mg Kg^−1^)	NO_3_^−^-N (mg Kg^−1^)	Soluble N (mg Kg^−1^)	Total N (%)	Total P (mg Kg^−1^)	Available P (mg Kg^−1^)	Total K (mg Kg^−1^)	Available K (mg Kg^−1^)
*Ag. adenophora* 1	28.78	5.81	256.2	3.38	3.85	658.2	1.182	26.5	0.121	261	0.18
*Ag. adenophora* 2	23.70	5.18	296.4	2.18	4.74	725.0	0.995	34.0	0.109	302	0.20
*Ag. adenophora* 3	27.37	5.90	287.3	2.21	3.45	394.4	0.929	18.1	0.117	174	0.10
*Ar. indica* 1	22.51	5.43	96.9	7.45	2.46	398.8	0.419	10.2	0.104	169	0.25
*Ar. indica* 2	18.69	5.37	98.3	2.93	1.76	348.6	0.426	14.9	0.115	260	0.24
*Ar. indica* 3	17.75	5.53	87.2	1.95	1.17	209.9	0.328	6.5	0.093	363	0.23
*I. cylindrica* 1	25.76	5.79	41.0	6.33	1.07	163.4	0.173	4.0	0.114	38	0.33
*I. cylindrica* 2	26.44	6.13	74.7	2.02	1.43	72.7	0.324	12.1	0.029	155	0.44
*I. cylindrica* 3	27.51	5.81	81.9	3.37	1.15	145.2	0.299	8.5	0.110	169	0.29

### Composition and biodiversity of bacterial and fungal communities

**Figure 2:**
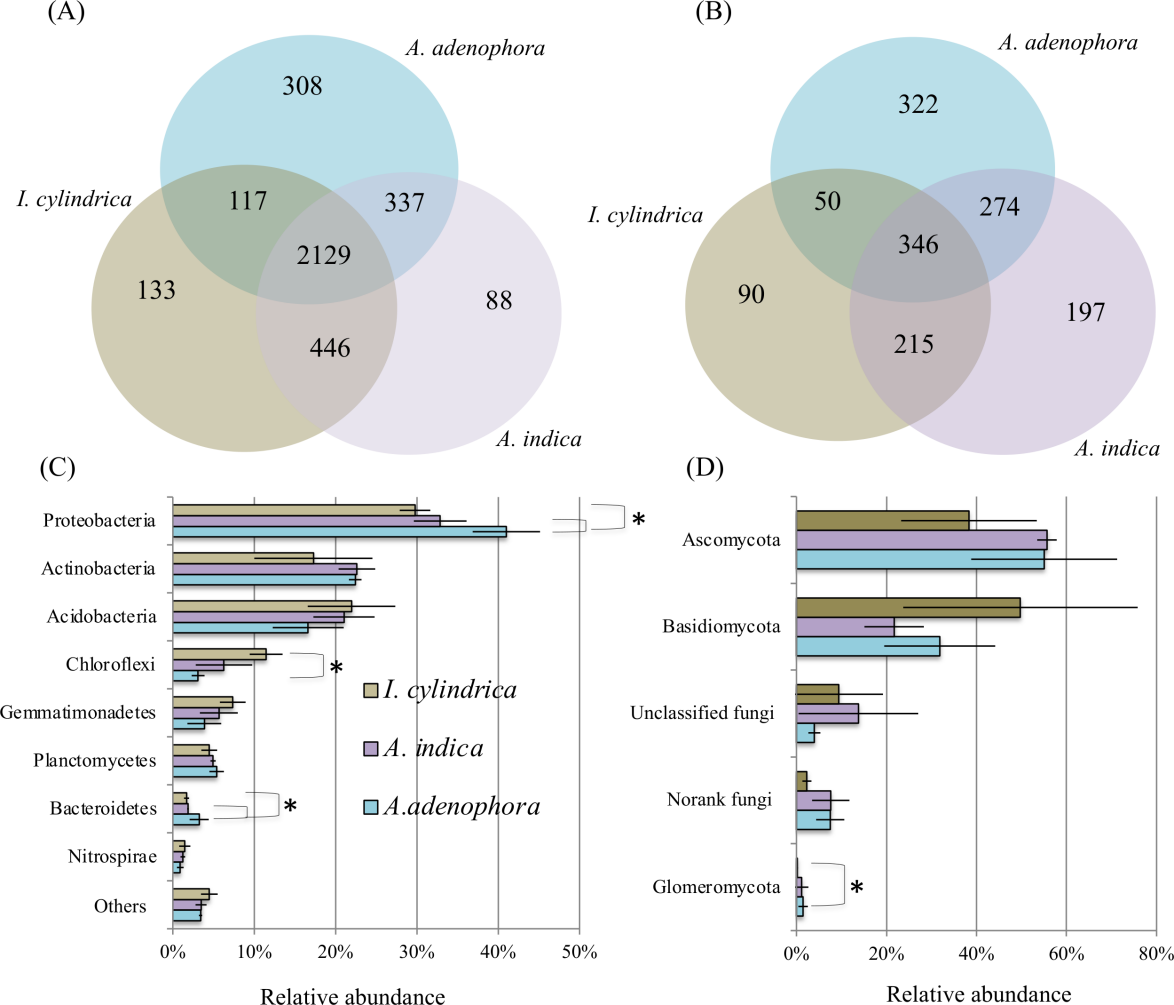
Diagrams of Venn (A & B) and phylum composition (C & D) of the rhizosphere soil microbiotas of invasive weed *Ageratina adenophora*, and native plant species *Artemisia indica* and *Imperata cylindrica*. (A & B) showing the occurrence of bacterial (A) and fungal (B) OTUs identified using 16S rRNA gene and ITS fragment sequencing. (C & D) showing the phylum compositions of bacterial (C) and fungal (D) communities. The symbol labels in (C) apply to (D). An asterisk (*) indicates significant difference in phylum relative percentage abundance.

16S rRNA and ITS amplicon sequences (Table 2) retrieved from the nine rhizosphere samples of *A. adenophora* and the native species *A. indica* and *I. cylindrica* (three replicas for each) were used to characterize the composition and biodiversity of the bacterial and fungal communities. A total of 46,364 to 66,570 high-quality 16S rRNA sequence reads and 30,659 to 43,822 fungal ITS sequence reads were retrieved from individual samples taken from the nine rhizosphere soil samples. Phylogenetic analyses showed that the sequences retrieved from each of the nine samples belonged to 1,648 and 2,531 bacterial and archaeal OTUs, respectively, and 198 to 665 fungal OTUs, respectively (Figs. 2A and 2B). Chao1 analyses (Table 2) estimated that individually, the RSMs contained 1,860 and 2,842 bacterial and archaeal OTUs, respectively, with a coverage range of 0.98 to 0.99, and 231 to 739 fungal OTUs with a coverage of 0.99, demonstrating that the sequencing depths used in this study covered the diversity of the RSMs from the three plants. Archaeal sequences only accounted for 0–0.05% of the 16S rRNA sequences obtained from individual soil sample. They were not investigated further in this study.

**Table 2:** Biodiversity index and similarity of bacterial and fungal communities in the rhizosphere soils of weed *Ageratina adenophora*, and native plant species *Artemisia indica* and *Imperata cylindrica*.

Sample description	Number of RSeq*		Number of HQSeq*		Number of OTUs		Chao1 richness		Shannon index		Coverage values	
	Fungi	Bacteria	Fungi	Bacteria	Fungi	Bacteria	Fungi	Bacteria	Fungi	Bacteria	Fungi	Bacteria
*Ag. adenophora* 1	62764	44556	56875	43822	2202	617	2477	671	6.23	4.14	0.99	0.99
*Ag. adenophora 2*	60046	39626	52712	39035	2143	592	2455	643	6.24	4.25	0.99	0.99
*Ag. adenophora 3*	64165	33607	57701	33037	2198	497	2519	554	6.13	3.83	0.99	0.99
*Ar. indica 1*	71454	44760	65216	43945	2120	665	2482	739	6.00	4.75	0.99	0.99
*Ar. indica 2*	50721	30909	46364	30659	1655	435	2039	494	5.72	3.64	0.99	0.99
*Ar. indica 3*	73284	35991	66570	35504	2531	523	2843	631	6.21	3.51	0.99	0.99
*I. cylindrica 1*	61819	38371	57131	38115	1648	198	1860	231	5.48	1.84	0.99	0.99
*I. cylindrica 2*	67419	33561	61994	35381	2027	262	2264	298	6.04	2.95	0.99	0.99
*I. cylindrica 3*	51598	44432	46368	43788	2275	576	2598	660	6.25	3.71	0.98	0.99

**Notes.**

* RSeq represents raw sequences; HQSeq represents high quality sequences.

The composition of the bacterial community in the RSM of *A. adenophora* differed from those in the RSM of the two native species. At the phylum level, the 16S rRNA gene sequences identified from the nine rhizosphere samples belonged to members of 37 divisions and consisted of 34 bacterial phyla. The majority were members of the phyla Proteobacteria, Actinobacteria, Acidobacteria, Chloroflexi, Gemmatimonadetes, Planctomycetes, Bacteroidetes, and Nitrospirae, where each of their mean percentage abundances in all soil samples were >1.20% (Fig. 2C). The *A. adenophora* RSM contained a higher percentage abundance of members of Proteobacteria (*P* = 0.05) and Bacteroidetes (*P* = 0.05) than those of *A. indica* and *I. cylindrica*, and a lower (*P* = 0.05) percentage abundance of Chloroflexi than that of *I. cylindrica* (Fig. 2C).

29%–81% of the 16S rRNA sequences identified for each of the nine RSM samples could be classified reliably at the genus level, where 497 individual genera or taxa were seen. Forty-seven of these were assessed to be relatively abundant, with each accounting for an average of at least 0.5% of the total sequences detected in the combined rhizosphere soil samples (Table S1). Of these, the RSM of *A. adenophora* contained a higher (*P* = 0.05) percentage abundance of Norank 480-2, Norank Subgroup 7, *Rhodoplanes*, uncultured *Xanthomonadales*, and *Variibacter,* and a lower (*P* = 0.05) percentage abundance of unclassified *Gemmatimonadaceae* than that of *A. indica*. It also possessed a higher (*P* = 0.05) abundance of the denitrifying *Haliangium*, *Acidibacter*, Norank 480-2, *Rhizomicrobium*, *Rhodoplanes*, unclassified *Comamonadaceae*, uncultured *Chitinophagaceae*, uncultured *Xanthomonadales*, and *Variibacter* and a lower (*P* = 0.05) percentage abundance of uncultured *Gemmatimonadaceae,* Norank TK10, *Anaeromyxobacter*, Norank Subgroup 7, Norank JG37-AG-4, Norank *Latescibacteria*, and unclassified *Gemmatimonadaceae* than *I. cylindrica* (Table S1).

As with the bacterial communities, the fungal community of the *A. adenophora* RSM differed less from that of *A. indica* than from that of *I. cylindrica*. ITS sequences identified from the nine rhizosphere soil samples belonged to members of six fungal phyla. Most were members of Ascomycota and Basidiomycota (Fig. 2D), while the remainder were members of the phyla Glomeromycota, Chytridiomycota, Blastocladiomycota, and Entomophthoromycota. The percentage abundance of Glomeromycota in the RSM of *A. adenophora* was higher (*P* = 0.05) than that in the RSM of *I. cylindrica* (Fig. 2D). However the percentage abundance was not significantly (*P* > 0.05) different than those of the other phyla detected between the RSM of *A. adenophora* and those of either of the two native species. The identified fungal ITS sequences belonged to 348 genera. Thirty-five were assessed to be relatively abundant, with each accounting for an average of at least 0.5% of the total fungal ITS sequences detected in all the rhizosphere soil samples (Table S2). The RSM of *A. adenophora* contained a higher (*P* = 0.05) percentage abundance of *Penicillium* and a lower (*P* = 0.05) percentage abundances of *Fusarium* and *Clavaria* than that of *A. indica,* and a higher (*P* ≤ 0.05) percentage abundance of *Mortierella*, *Penicilium*, *Cladophialophora*, *Metarhizium*, *Exophiala*, *Nectria*, *Pyrenochaeta*, *Oliveonia*, *Acremonium*, and *Hypomyces* and a lower (*P* ≤ 0.05) percentage abundance of *Gilophorus*, *Pyrenula*, and *Troposporella* than that of *I. cylindrica* (Table S2).

### Function and expression of unigenes identified in the rhizosphere microbiomes of *A*. *adenophora* and the two native species

Approximately 314 million raw paired-end RNA sequence reads were retrieved from the total rhizosphere soil samples of the three plants. Between 97 and 112 million high-quality reads were retrieved for each of the rhizosphere RNA samples. After the removal of rRNA reads, the rhizosphere soils of *A. adenophora*, *A. indica*, and *I. cylindrica* contained 6,922,157, 4,263,665, and 3,203,005 cDNA reads, respectively, for ORF prediction. Furthermore, 75,913, 55,826, and 39,458 unigenes were identified in their rhizosphere soils, respectively.

**Figure 3:**
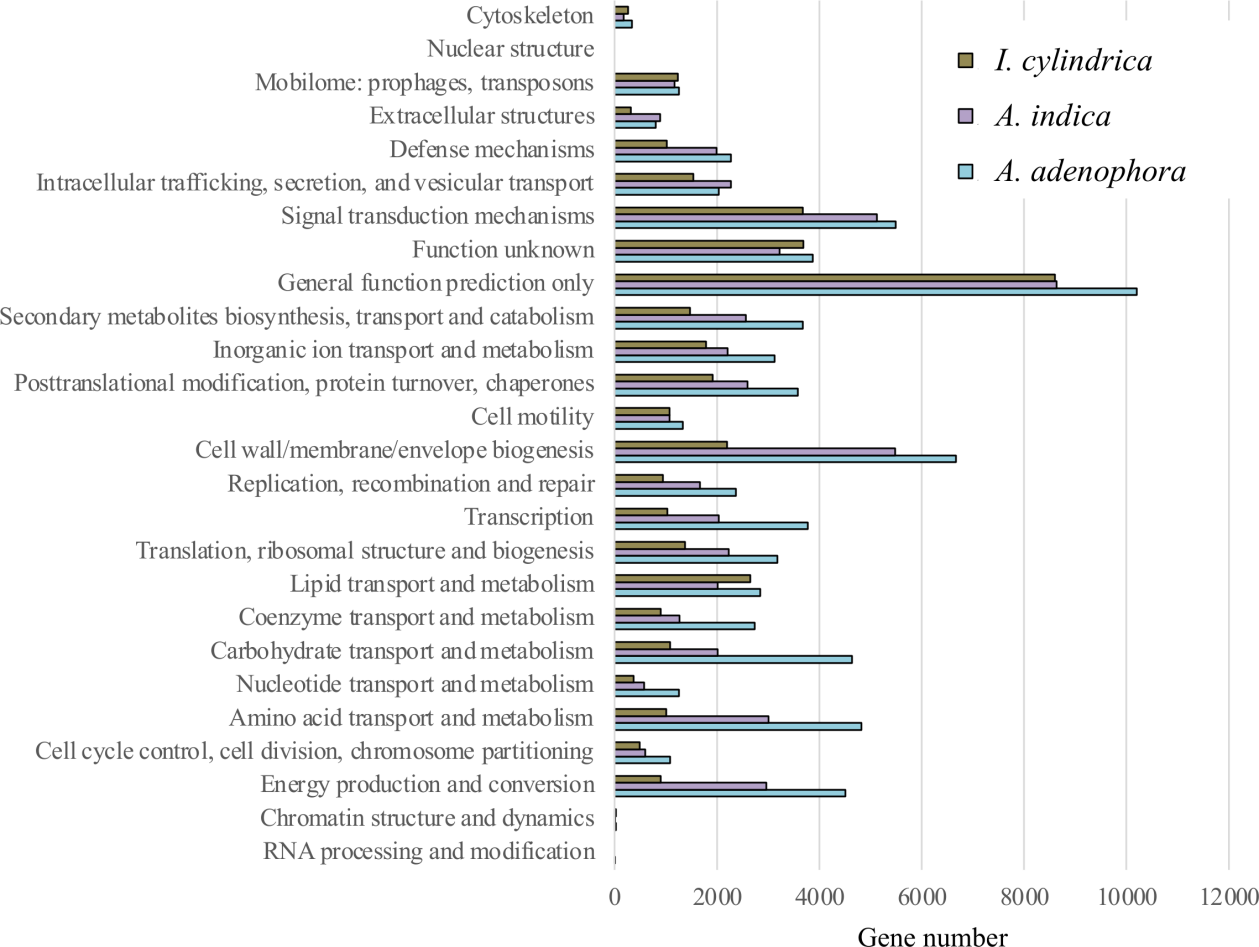
Expression of functional genes in the rhizosphere soils of weed *Ageratina adenophora*, and native plant species *Artemisia indica* and *Imperata cylindrica* based on cluster of orthologous groups.

The expression numbers of the unigenes classified with COG function in each rhizosphere soil sample are shown in Fig. 3. Of those with known functions the abundant were: genes encoding polypeptides associated with cell wall, membrane, and envelope biogenesis; signal transduction mechanisms; amino acid transport and metabolism; energy production and conversion; posttranslational modification, protein turnover, and chaperones; carbohydrate transport and metabolism; secondary metabolites biosynthesis, transport, and catabolism; and lipid transport and metabolism (an average of between 2,505 and 4,782 genes each for all samples). The genes encoding polypeptides associated with inorganic transport and metabolism, transcription and translation, ribosomal structure, and biogenesis were less abundant (each contributing approximately 2,263 to 2,373 genes).

The expression levels of functional genes in the RSM of *A. adenophora* differed from those in the RSMs of the two native plant species. Thus, the RSM of *A. adenophora* displayed 1.2 and 3.0 times the expression levels of genes encoding for cell wall, membrane, and envelope biogenesis; 1.6 and 4.8 times those of genes encoding amino acid transport and metabolism; 1.5 and 5.0 times those of genes encoding energy production and conversion; 2.3 and 4.3 times those of genes encoding carbohydrate transport and metabolism; 1.4 and 2.5 times those of genes encoding secondary metabolite biosynthesis, transport, and catabolism; 1.9 and 3.7 times those of genes encoding transcription; 1.4 and 2.3 times those of genes encoding translation, ribosomal structure, and biogenesis; 1.4 and 2.5 times those of genes encoding replication, recombination, and repair; 2.2 and 3.0 times those of genes encoding coenzyme transport and metabolism; and 2.2 and 3.4 times those of genes encoding nucleotide transport and metabolism compared to those of genes expressed in the RSMs of *A. indica* and *I. cylindrica*, respectively (Fig. 3).

### Phylogenetic classification and relative abundance of functional genes identified in the rhizosphere microbiomes of *A. adenophora* and the two native plant species

**Figure 4:**
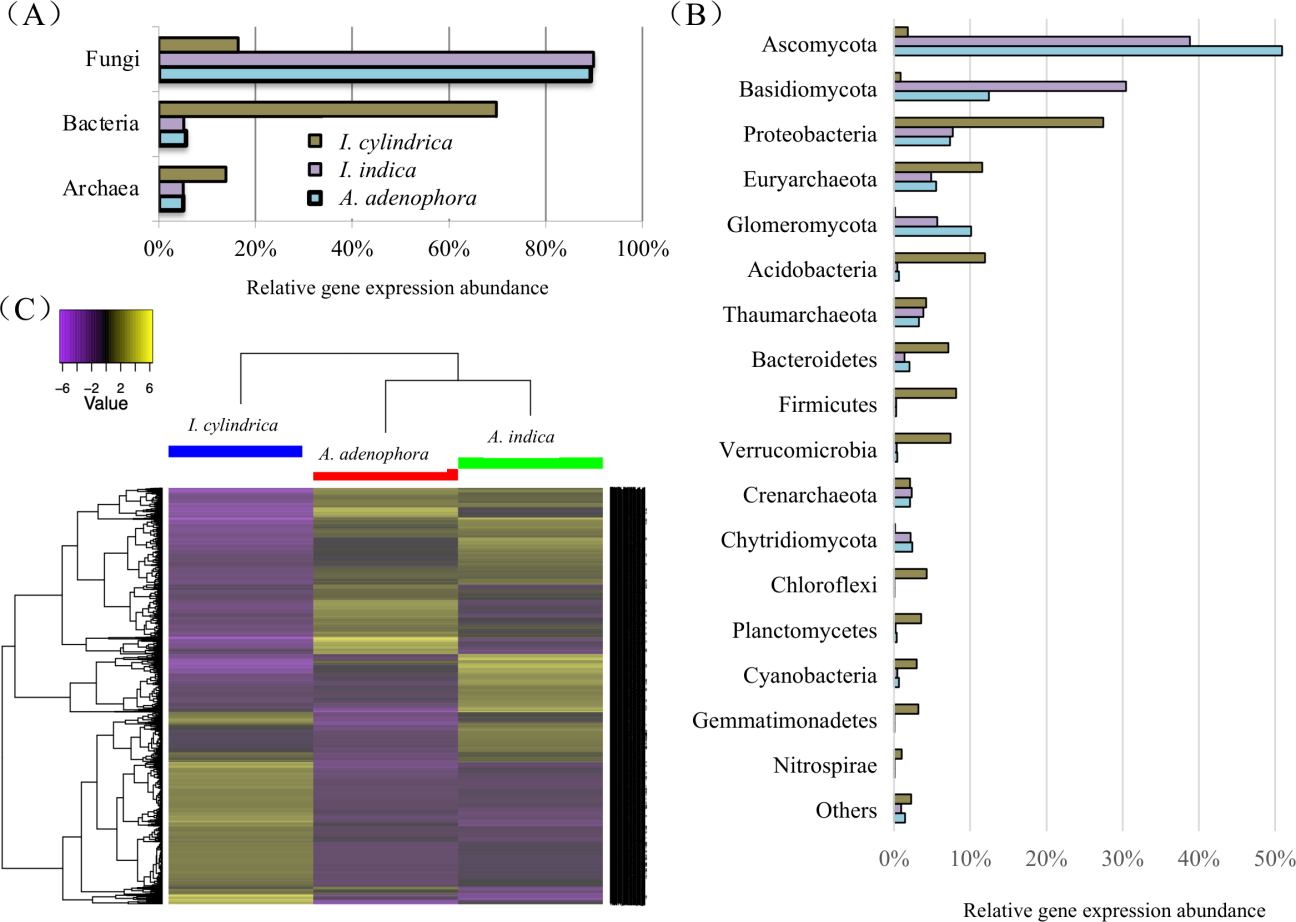
Taxonomic classification and heatmap of unigenes identified in the rhizosphere soils of weed *Ageratina adenophora*, and native plant species *Artemisia indica* and *Imperata cylindrica*. (A & B) Taxonomic classification at kingdom (A) and phylum (B) levels and relative abundances of the classifiable unigenes. (C) Heatmap of unigenes showing composition and relative abundances of the unigenes. The symbol labels in (A) apply to (B).

The taxonomic relationships of the unigenes identified in the *A. adenophora* RSM were similar to those identified in the *A. indica* RSM, but differed markedly from those in the *I. cylindrica* RSM. Fungi contributed to most of the total genes expressed in the RSMs of both *A. adenophora* and *A. indica* at the kingdom level, while those from *Bacteria* and *Archaea* accounted for the majority of the genes expressed in the *I. cylindrica* RSM (Fig. 4A). Thus, fungal members from the phyla Ascomycota, Basidiomycota, and Glomeromycota accounted for the majority of the genes expressed in the RSMs of *A. adenophora* and *A. indica*, while prokaryote members belonging to Proteobacteria, Euryarchaeota, Acidobacteria, Bacteroidetes, Firmicutes, Verrucomirobia, and Cyanobacteria were the major contributors in the *I. cylindrica* RSM (Fig. 4B). Furthermore, at the gene level, the RSM of *A. adenophora* was closer in composition and relative expression level of unigenes to those in the RSM of *A. indica* than to those in the RSM of *I. cylindrica* (Fig. 4C).

### Expression of nitrogen metabolism associated genes in the rhizosphere microbiomes of *A. adenophora* and both native plant species

**Figure 5:**
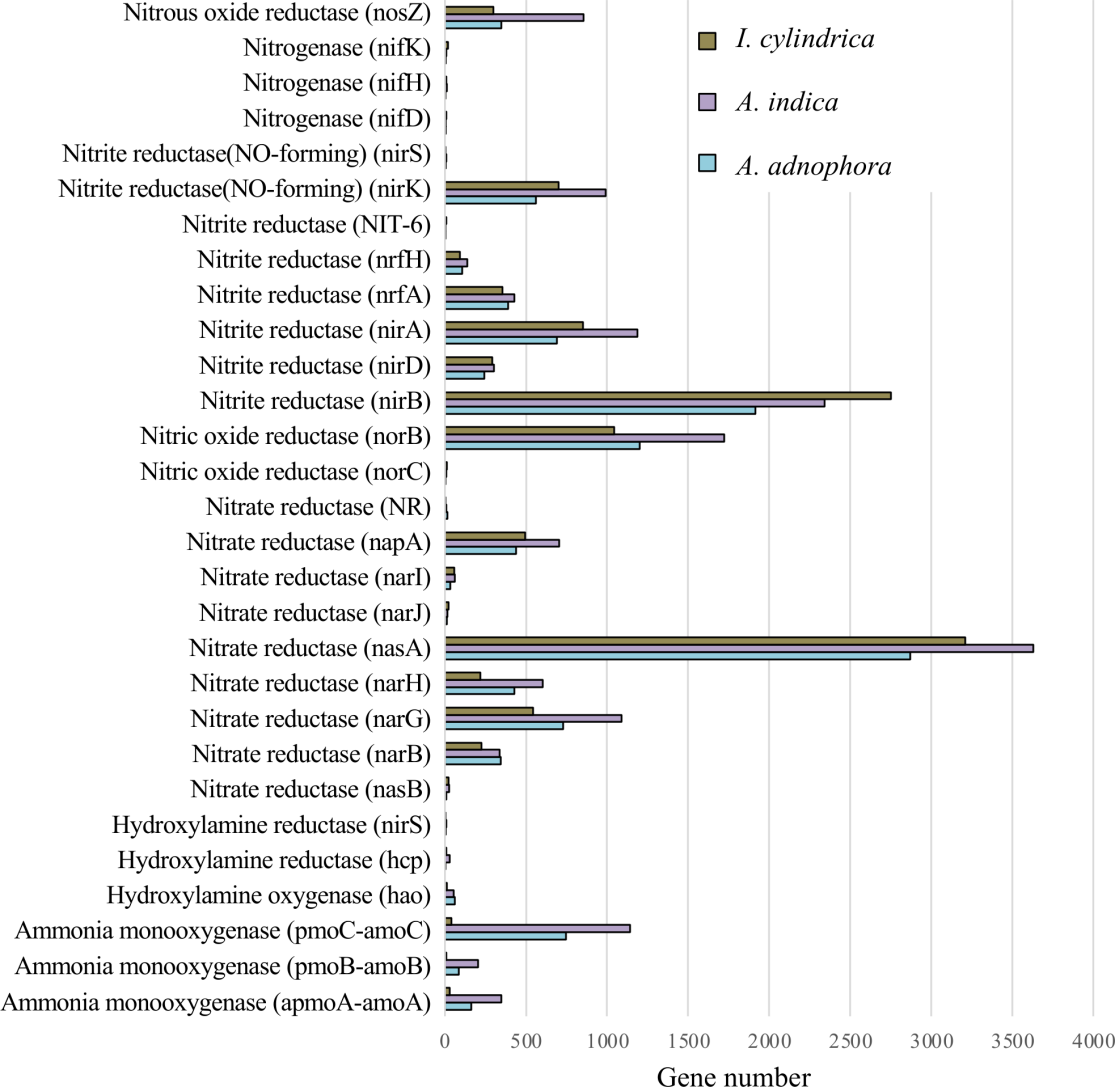
Expression of nitrogen metabolism genes in the rhizosphere soils of invasive weed *Ageratina adenophora*, and native plant species *Artemisia indica* and *Imperata cylindrica*.

The expression profiles of genes involved in nitrogen metabolism in the RSM of *A. adenophora* were similar to those in the RSMs of both native plant species (Fig. 5). Thus, the read numbers of genes encoding nitrogenase, nitrous oxide reductase, nitrite reductase, nitric oxide reductase, hydroxylamine reductase, hydroxylamine oxygenase, and hydroxylamine monooxygenase in the RSM of *A. adenophora* did not differ substantially from those in either of the two native plant species (Fig. 5). One exception was the nitrate reductase encoding gene, whose read number was markedly higher in the *A. adenophora* RSM.

## Discussion

We compared the compositional and metabolic characteristics of the RSM of the highly invasive weed *A. adenophora* against those of two native species to elucidate the effects of *A. adenophora* root exudates on the composition and metabolism of its RSM and assessed the role of bacterial and fungal communities present in its nutrient cycles. We found that the composition of the bacterial and fungal communities of the *A. adenophora* RSM varied in differing degrees to those of the two native species (Tables S1& S2, Figs. 2C& 2D). More compositional differences were observed in the RSM of *I. cylindrica* in the uninvaded area than in that of *A. indica* co-existing with *A. adenophora*. The RSM of *A. adenophora* had a higher (*P* ≤ 0.05) percentage of bacterial Norank 480-2, *Rhodoplanes*, uncultured *Xanthomonadales*, *Variibacter,* and fungal *Penicillium* than those of the native plant species. In an earlier study, Zhao et al. (2019) found members of the genera Gp6 (*Acidobacteria*), together with *Sphingomonas*, and *Spartobacteria* contributed most to the dissimilarities between rhizosphere soils of the weed *A. adenophora* and other different native plant species. Such a striking discrepancy probably reflects differences in the native plant species and soil types used in the two studies.

Our 16S rRNA gene and ITS fragment sequencing (Figs. 2C & 2D) and transcriptome data (Fig. 4B) are in agreement in showing fungi belonging to phyla Ascomycota and Basidiomycota, and bacteria belonging to phyla Proteobacteria, Acidobacteria, and Bacteroidetes are important players in the RSMs of *A. adenophora* and the two native plant species. The RSM fungal community of *A. adenophora* is thought to play a major role in its nutrition. The fungal community contributed 89.3% of the expressed genes (Fig. 4A) and most (71.2%) were from members of Ascomycota and Basidiomycota (Fig. 4B). Of these, Ascomycota constituted more than half of ITS total reads (55%) and expressed genes (51%) in the *A. adenophora* RSM. Saprotrophic fungi are known to be highly abundant active organisms in the rhizosphere, where they metabolize root exudates (Denef et al., 2007; Bueé et al., 2009) even more successfully than the rhizosphere soil bacteria under certain conditions (Rudnick, Van Veen & De Boer, 2015).

Glomeromycota contributed 10.1% of the genes expressed in the *A. adenophora* RSM, which was substantially higher than that expressed in the RSMs of both *A. indica* (5.7%) and *I. cylindrica* (0.1%) (Fig. 4B), and so the RSM of *A. adenophora* had more (*P* = 0.05) of these symbiotic arbuscular mycorrhizal (AM) fungi than did that of *I. cylindrica* (Fig. 2D). Therefore, it is likely they play a positive role in successful *A. adenophora* invasion by enhancing the provision of N and P, especially if there are limited amounts in the soil, and in protecting it from parasite attack at the roots (*Johnson et al., 2010*). The supply of nitrogen is known to play an important role in exotic plant invasion (*Bajpai & Inderjit, 2013; Perkin & Nowak, 2013*) especially since the gene expression profiles of enzymes associated with nitrogen fixation and transformation metabolism in the *A. adenophora* RSM were similar to those of both *A. indica* and *I. cylindrica* (Fig. 5). These data suggest that attracting and stimulating the metabolic activity of Glomeromycota enhances the invasive capabilities of *A. adenophora*, and is the first metabolic evidence to support an important role for AM fungi in *A. adenophora* invasion.

We showed that there were greater similarities in the composition and expression levels of functional genes in the *A. adenophora* and *A. indica* RSMs than in *I. cylindrica* (Figs. 3 and 4A, 4B, & 4C). Such compositional and metabolic similarities in the RSMs may explain why *A. indica* can withstand invasion attempts by *A. adenophora,* as shown by their coexistence at one site for at least 8 years. Higher levels of expression of genes associated with cell wall, membrane, and envelope biogenesis; amino acid transport and metabolism; energy production and conversion; carbohydrate transport and metabolism; secondary metabolite biosynthesis, transport, and catabolism; transcription; translation; ribosomal structure and biogenesis; replication, recombination, and repair; coenzyme transport and metabolism; and nucleotide transport and metabolism were seen in the RSM of *A. adenophora* than in either the RSMs of *A. indica* and especially *I. cylindrica* (Fig. 3). Such enhancement of expression of these genes would suggest that the *A. adenophora* RSM is more metabolically active than those of either native species.

Invasion by *A. adenophora* did not affect gene expression levels associated with microbial nitrogen fixation, ammonia oxidation, and nitrite and nitrate reduction in the invaded soil. There was no evidence for the increased expression of N metabolic genes, which may also reflect high amounts of organic C (litter) present in the RSM of *A. adenophora* (Table 1), where its decomposition may release organic N in higher amounts than from either native plants, as reported for other invasive plant species (Ehrenfeld, Ravit & Elgersma, 2005; Hata, Kato & Kachi, 2012; Kaur et al., 2012; Meisner et al., 2012; Li et al., 2017). This is supported by evidence that more genes are associated with carbohydrate metabolism in the RSM of *A. adenophora* than in those of the native plant species. Interestingly, in their GeoChip study, Zhao et al. (2019) showed that the *A. adenophora* RSM had higher nitrogen metabolism gene expression levels than those found in the native plant species examined. Such differences may arise from the measurement techniques used. GeoChip assays quantify gene expression in terms of the total DNA, transcriptomic analysis measures gene expression levels as mRNA synthesis. Zhao et al. (2019) compared the rhizosphere soil of *A. adenophora* to that of a mixture of soil samples from seven native plant species, and we compared the rhizosphere soil of *A. adenophora* individually with those of two native plant species.

## Conclusions

In conclusion, the data obtained in this study suggests that the RSM of the weed, *A. Adenophora*, differs substantially in its bacterial and fungal communities to those of the two native plant species. The RSM of *A. adenophora* contained higher percentage abundances of Proteobacteria and Bacteroidetes than those of the native plant species. Increased expression levels of genes associated with metabolic activities of transport and metabolism of amino acids, coenzymes, nucleotides and carbohydrates; energy production and conversion; transcription; and eventual cell growth in the *A. adenophora* RSM play an important enhancing role in the nutritional metabolism of its rhizosphere soils, allowing it to invade a habitat already occupied by two native species, *A. indica* and *I. cylindrica.* The data also indicate that an increased level of gene expression in members of the fungal communities, especially the saprotrophic Ascomycota and Basidiomycota and the AM fungi Glomeromycota, played a role in its successful invasion.

##  Supplemental Information

10.7717/peerj.10844/supp-1 Supplemental Information 1 Relative abundance of the dominant (average relative abundance > 0.5%) bacterial genera identified in the rhizosphere soils of weed *Ageratina adenophora* and native plant species AGE: *Ageratina adenophora;* ART: *Artemisia indica;* IMP: *Imperata cylindrica.*

10.7717/peerj.10844/supp-2 Supplemental Information 2 Relative abundance of the dominant (average relative abundance > 0.5%) fungal genera identified in the rhizosphere soils (RSs) of weed *Ageratina adenophora and native plant species.* AGE: *Ageratina adenophora;* ART: *Artemisia indica;* IMP: *Imperata cylindrica.*

10.7717/peerj.10844/supp-3 Supplemental Information 3 Statistical analysis of raw physiochemical data in Table 1

## Additional Information and Declarations

### Data Availability

The following information was supplied regarding data availability: The 16S rRNA and ITS amplicon sequences are available at the NCBI Sequence Read Archive (SRA): PRJNA633422. The raw RNA sequence dataset is also available in the NCBI SRA: PRJNA638694. Additional raw data are available in the Supplemental Files.
