# Supplementary material for: Compositional and functional profiling of the rhizosphere microbiomes of the invasive weed Ageratina adenophora and native plants
Source: PeerJ. 2021 Mar 4;9:e10844. doi: 10.7717/peerj.10844 (PMC7937340; doi:10.7717/peerj.10844)
Supplement: Supplemental Information 1 — AGE: Ageratina adenophora; ART: Artemisia indica; IMP: Imperata cylindrica. [file peerj-09-10844-s001.docx]

| Genus name* | Relative abundance of *Ag. adenophora* | | | | Relative abundance of *Ar. indica* | | | | Relative abundance of *I. cylindrica* | | | | P value | |
| --- | --- | --- | --- | --- | --- | --- | --- | --- | --- | --- | --- | --- | --- | --- |
|  | Sample  1 | Sample  2 | Sample  3 | Mean | Sample  1 | Sample  2 | Sample  3 | Mean | Sample  1 | Sample  2 | Sample  3 | Mean | AGE vs  ART | AGE vs  IMP |
| Subgroup 6_norank | 8.23% | 3.47% | 4.46% | 5.39% | 6.51% | 3.76% | 4.18% | 4.82% | 5.12% | 6.04% | 4.23% | 5.13% | 0.8270 | 0.8270 |
| Unc *Acidobacteriaceae* (Subgroup 1) | 1.51% | 1.51% | 4.41% | 2.48% | 4.01% | 7.01% | 4.02% | 5.01% | 5.80% | 3.31% | 4.50% | 4.54% | 0.2750 | 0.1270 |
| Unc *Gaiellales* | 3.10% | 1.90% | 4.12% | 3.04% | 4.05% | 4.80% | 3.89% | 4.25% | 1.85% | 3.97% | 3.85% | 3.23% | 0.2750 | 0.8270 |
| Subgroup 2_norank | 0.63% | 1.42% | 3.44% | 1.83% | 1.36% | 5.01% | 5.18% | 3.85% | 9.80% | 1.70% | 2.84% | 4.78% | 0.2750 | 0.2750 |
| Unc *Gemmatimonadaceae* | 2.99% | 1.02% | 1.15% | 1.72% | 3.27% | 0.89% | 4.97% | 3.04% | 8.06% | 4.34% | 3.26% | 5.22% | 0.5130 | 0.0500 |
| *Bradyrhizobium* | 2.42% | 3.43% | 4.09% | 3.31% | 3.66% | 3.69% | 2.96% | 3.44% | 1.65% | 3.14% | 2.94% | 2.58% | 0.8270 | 0.2750 |
| Unc *Nitrosomonadaceae* | 3.69% | 0.83% | 1.92% | 2.15% | 2.87% | 1.27% | 2.71% | 2.28% | 6.72% | 3.31% | 2.12% | 4.05% | 0.8270 | 0.2750 |
| DA111_norank | 1.11% | 1.63% | 3.11% | 1.95% | 1.59% | 4.69% | 2.88% | 3.05% | 2.88% | 1.93% | 4.29% | 3.03% | 0.5130 | 0.2750 |
| Uncl *Rhizobiales* | 2.35% | 1.97% | 3.43% | 2.58% | 2.56% | 4.12% | 2.16% | 2.95% | 0.96% | 2.74% | 2.60% | 2.10% | 0.5130 | 0.8270 |
| *Acidothermus* | 0.75% | 1.31% | 3.89% | 1.98% | 2.12% | 3.97% | 2.37% | 2.82% | 0.78% | 1.46% | 3.27% | 1.83% | 0.2750 | 0.8270 |
| *Haliangium* | 2.99% | 2.61% | 2.62% | 2.74% | 2.91% | 1.59% | 1.52% | 2.01% | 0.88% | 1.97% | 1.99% | 1.61% | 0.2750 | 0.0500 |
| *Gemmatimonas* | 2.93% | 1.28% | 1.05% | 1.75% | 2.65% | 1.08% | 1.66% | 1.80% | 0.78% | 1.57% | 1.79% | 1.38% | 0.8270 | 0.8270 |
| Unc *Acidimicrobiales* | 1.60% | 1.06% | 1.77% | 1.48% | 1.29% | 1.68% | 1.65% | 1.54% | 1.31% | 2.11% | 1.87% | 1.77% | 0.8270 | 0.2750 |
| *Acidibacter* | 1.49% | 3.20% | 1.74% | 2.14% | 1.43% | 2.43% | 1.07% | 1.64% | 0.54% | 0.39% | 1.01% | 0.65% | 0.2750 | 0.0500 |
| TK10_norank | 1.08% | 0.36% | 0.94% | 0.79% | 1.25% | 0.93% | 1.90% | 1.36% | 2.82% | 2.13% | 1.67% | 2.21% | 0.2750 | 0.0500 |
| Unc *Planctomycetaceae* | 1.23% | 1.05% | 1.82% | 1.37% | 0.90% | 0.99% | 1.78% | 1.23% | 1.33% | 1.72% | 1.67% | 1.58% | 0.2750 | 0.5130 |
| 480-2_norank | 1.86% | 2.06% | 1.72% | 1.88% | 1.52% | 1.59% | 0.71% | 1.27% | 0.10% | 1.25% | 0.75% | 0.70% | 0.0500 | 0.0500 |
| *Anaeromyxobacter* | 0.95% | 0.16% | 0.24% | 0.45% | 1.05% | 0.43% | 1.51% | 1.00% | 3.23% | 2.42% | 1.31% | 2.32% | 0.1270 | 0.0500 |
| RB41_norank | 0.66% | 1.35% | 1.50% | 1.17% | 0.52% | 0.16% | 2.18% | 0.95% | 1.45% | 1.23% | 1.84% | 1.51% | 0.5130 | 0.5130 |
| *Nitrospira* | 1.44% | 0.76% | 0.56% | 0.92% | 1.03% | 1.60% | 1.07% | 1.23% | 2.32% | 1.30% | 0.81% | 1.47% | 0.2750 | 0.2750 |
| WD2101 soil group_norank | 1.89% | 0.76% | 0.76% | 1.14% | 2.14% | 1.37% | 1.11% | 1.54% | 0.41% | 0.70% | 1.14% | 0.75% | 0.2750 | 0.2750 |
| *Rhizomicrobium* | 1.15% | 2.93% | 1.61% | 1.90% | 1.12% | 1.45% | 0.50% | 1.02% | 0.43% | 0.23% | 0.71% | 0.46% | 0.1270 | 0.0500 |
| Uncl *Xanthobacteraceae* | 0.84% | 0.55% | 1.81% | 1.07% | 1.52% | 1.68% | 0.92% | 1.37% | 0.57% | 1.36% | 0.77% | 0.90% | 0.5130 | 0.8270 |
| Uncl *Micromonosporaceae* | 0.57% | 1.06% | 0.86% | 0.83% | 1.05% | 1.46% | 1.46% | 1.32% | 0.54% | 1.25% | 1.52% | 1.10% | 0.1270 | 0.5130 |
| Candidatus *Solibacter* | 0.56% | 0.84% | 1.44% | 0.95% | 1.03% | 1.66% | 0.82% | 1.17% | 1.20% | 0.74% | 1.15% | 1.03% | 0.5130 | 0.8270 |
| *Gaiella* | 1.13% | 0.39% | 0.85% | 0.79% | 1.13% | 1.48% | 0.95% | 1.19% | 0.58% | 1.32% | 0.87% | 0.92% | 0.2750 | 0.5130 |
| *Bryobacter* | 0.46% | 0.94% | 1.56% | 0.99% | 0.77% | 1.15% | 0.86% | 0.93% | 1.37% | 0.51% | 1.01% | 0.97% | 0.8270 | 0.8270 |
| Subgroup 7_norank | 0.78% | 0.81% | 0.76% | 0.78% | 0.30% | 0.40% | 0.73% | 0.48% | 1.36% | 2.16% | 0.86% | 1.46% | 0.0500 | 0.0500 |
| *Solirubrobacter* | 2.16% | 0.83% | 0.77% | 1.25% | 1.78% | 0.30% | 0.65% | 0.91% | 0.02% | 0.67% | 0.95% | 0.55% | 0.8270 | 0.8270 |
| GR-WP33-30_norank | 0.84% | 0.52% | 0.98% | 0.78% | 0.64% | 1.06% | 0.83% | 0.84% | 1.23% | 0.77% | 0.83% | 0.94% | 0.8270 | 0.8270 |
| KD4-96_norank | 1.02% | 0.23% | 0.97% | 0.74% | 1.18% | 0.36% | 0.92% | 0.82% | 0.48% | 1.51% | 0.99% | 0.99% | 0.8270 | 0.5130 |
| SC-I-84_norank | 0.48% | 0.58% | 1.10% | 0.72% | 1.17% | 0.89% | 1.00% | 1.02% | 0.39% | 1.02% | 0.99% | 0.80% | 0.2750 | 0.8270 |
| *Rhodoplanes* | 1.07% | 1.19% | 0.83% | 1.03% | 0.82% | 0.75% | 0.67% | 0.74% | 0.20% | 0.64% | 0.70% | 0.51% | 0.0500 | 0.0500 |
| *Reyranella* | 0.81% | 0.74% | 0.61% | 0.72% | 1.13% | 0.68% | 0.55% | 0.79% | 0.52% | 0.98% | 0.55% | 0.68% | 0.8270 | 0.5130 |
| *Streptomyces* | 0.59% | 2.06% | 0.51% | 1.05% | 1.00% | 0.81% | 0.59% | 0.80% | 0.18% | 0.30% | 0.53% | 0.34% | 0.5130 | 0.1270 |
| YNPFFP1_norank | 0.43% | 0.53% | 0.90% | 0.62% | 0.63% | 0.58% | 1.08% | 0.76% | 0.28% | 0.77% | 1.18% | 0.74% | 0.2750 | 0.8270 |
| *Pseudonocardia* | 1.01% | 0.76% | 0.59% | 0.79% | 1.26% | 0.23% | 0.79% | 0.76% | 0.05% | 0.64% | 0.96% | 0.55% | 0.8270 | 0.5130 |
| ABS-19_norank | 0.42% | 0.09% | 0.19% | 0.23% | 1.16% | 3.75% | 0.12% | 1.68% | 0.05% | 0.11% | 0.16% | 0.11% | 0.2750 | 0.2750 |
| JG37-AG-4_norank | 0.00% | 0.00% | 0.01% | 0.00% | 0.01% | 0.04% | 0.62% | 0.22% | 3.48% | 1.25% | 0.48% | 1.74% | 0.1270 | 0.0500 |
| Unc *Xanthobacteraceae* | 0.45% | 0.19% | 0.79% | 0.48% | 0.22% | 0.45% | 0.91% | 0.52% | 0.88% | 0.76% | 1.24% | 0.96% | 0.5130 | 0.1270 |
| Uncl *Comamonadaceae* | 0.68% | 1.67% | 0.68% | 1.01% | 0.75% | 0.34% | 0.44% | 0.51% | 0.13% | 0.29% | 0.49% | 0.30% | 0.2750 | 0.0500 |
| Latescibacteria_norank | 0.21% | 0.01% | 0.13% | 0.12% | 0.36% | 0.04% | 0.70% | 0.37% | 1.94% | 1.62% | 0.39% | 1.31% | 0.2750 | 0.0500 |
| Unc *Chitinophagaceae* | 0.59% | 1.02% | 0.97% | 0.86% | 0.65% | 0.65% | 0.43% | 0.58% | 0.09% | 0.45% | 0.41% | 0.32% | 0.2750 | 0.0500 |
| Unc *Xanthomonadales* | 0.92% | 1.21% | 1.26% | 1.13% | 0.41% | 0.45% | 0.23% | 0.36% | 0.13% | 0.36% | 0.24% | 0.24% | 0.0500 | 0.0500 |
| Uncl *Gemmatimonadaceae* | 0.39% | 0.10% | 0.37% | 0.29% | 0.65% | 0.72% | 0.86% | 0.74% | 0.57% | 0.61% | 0.83% | 0.67% | 0.0500 | 0.0500 |
| *Variibacter* | 1.03% | 0.87% | 0.65% | 0.85% | 0.59% | 0.32% | 0.41% | 0.44% | 0.23% | 0.38% | 0.38% | 0.33% | 0.0500 | 0.0500 |
| *Pedomicrobium* | 0.70% | 0.46% | 0.64% | 0.60% | 0.60% | 0.62% | 0.36% | 0.53% | 0.20% | 0.82% | 0.42% | 0.48% | 0.2750 | 0.5130 |

*Unc represents uncultured; Uncl represents unclassified.
