# Supplementary material for: Compositional and functional profiling of the rhizosphere microbiomes of the invasive weed Ageratina adenophora and native plants
Source: PeerJ. 2021 Mar 4;9:e10844. doi: 10.7717/peerj.10844 (PMC7937340; doi:10.7717/peerj.10844)
Supplement: Supplemental Information 2 — AGE: Ageratina adenophora; ART: Artemisia indica; IMP: Imperata cylindrica. [file peerj-09-10844-s002.docx]

| Genus name | Relative abundance in the RSs of  *Ag. adenophora* | | | | Relative abundance in the RSs of  *Ar. indica* | | | | Relative abundance in the RSs of  *I. cylindrica* | | | | P value | |
| --- | --- | --- | --- | --- | --- | --- | --- | --- | --- | --- | --- | --- | --- | --- |
|  | Sample 1 | Sample 2 | Sample 3 | Mean | Sample 1 | Sample 2 | Sample 3 | Mean | Sample 1 | Sample 2 | Sample 3 | Mean | AGE  vs  ART | AGE  vs  IMP |
| *Purpureocillium* | 20.73% | 5.04% | 7.33% | 11.03% | 4.32% | 21.72% | 8.58% | 11.54% | 12.35% | 14.76% | 20.42% | 15.85% | 0.8270 | 0.5130 |
| *Gliophorus* | 0.00% | 0.00% | 0.00% | 0.00% | 0.00% | 0.00% | 0.10% | 0.03% | 70.12% | 27.56% | 0.04% | 32.57% | 0.3170 | 0.0370 |
| Unclassified fungi | 3.69% | 2.66% | 5.60% | 3.98% | 4.90% | 5.47% | 30.91% | 13.76% | 1.02% | 5.18% | 21.86% | 9.35% | 0.2750 | 0.8270 |
| *Ramariopsis* | 0.00% | 1.53% | 21.11% | 7.55% | 0.00% | 0.01% | 2.70% | 0.90% | 0.00% | 23.67% | 3.75% | 9.14% | 0.6580 | 0.6580 |
| *Mortierella* | 7.05% | 4.14% | 11.04% | 7.41% | 10.59% | 9.63% | 2.28% | 7.50% | 1.41% | 1.97% | 3.51% | 2.29% | 0.8270 | 0.0500 |
| *Tomentella* | 10.20% | 0.47% | 0.05% | 3.57% | 4.78% | 17.33% | 0.01% | 7.37% | 0.01% | 0.08% | 0.00% | 0.03% | 0.8270 | 0.1270 |
| *Chaetomium* | 5.13% | 0.95% | 2.32% | 2.80% | 3.69% | 4.45% | 1.15% | 3.09% | 1.15% | 0.94% | 2.21% | 1.43% | 0.8270 | 0.2750 |
| *Leohumicola* | 0.02% | 0.06% | 0.37% | 0.15% | 0.03% | 0.07% | 15.18% | 5.09% | 0.07% | 0.29% | 5.86% | 2.07% | 0.5130 | 0.2750 |
| *Penicillium* | 1.98% | 10.21% | 2.71% | 4.96% | 1.88% | 1.14% | 1.04% | 1.35% | 0.08% | 0.22% | 1.43% | 0.58% | 0.0500 | 0.0500 |
| *Fusarium* | 1.54% | 1.26% | 1.64% | 1.48% | 3.58% | 2.71% | 1.67% | 2.65% | 1.04% | 2.15% | 1.85% | 1.68% | 0.0500 | 0.5130 |
| *Cryptococcus* | 3.60% | 0.96% | 1.17% | 1.91% | 7.56% | 1.06% | 1.25% | 3.29% | 0.02% | 0.35% | 1.47% | 0.61% | 0.5130 | 0.2750 |
| *Clavaria* | 0.16% | 0.07% | 1.04% | 0.42% | 4.71% | 1.06% | 4.37% | 3.38% | 0.10% | 0.08% | 5.27% | 1.82% | 0.0500 | 0.8270 |
| *Ramaria* | 0.00% | 16.37% | 0.01% | 5.46% | 0.00% | 0.00% | 0.00% | 0.00% | 0.00% | 0.00% | 0.00% | 0.00% | 0.2460 | 0.1210 |
| *Cladophialophora* | 2.66% | 3.03% | 1.49% | 2.40% | 1.61% | 0.87% | 0.59% | 1.02% | 0.03% | 0.46% | 0.74% | 0.41% | 0.1270 | 0.0500 |
| *Metarhizium* | 3.18% | 2.30% | 1.07% | 2.18% | 2.73% | 0.73% | 0.15% | 1.20% | 0.04% | 1.03% | 0.12% | 0.40% | 0.2750 | 0.0500 |
| *Suillus* | 0.00% | 0.00% | 10.93% | 3.64% | 0.00% | 0.00% | 0.00% | 0.00% | 0.00% | 0.00% | 0.00% | 0.00% | 0.1210 | 0.1210 |
| *Hygrocybe* | 0.00% | 0.00% | 1.96% | 0.65% | 0.00% | 0.00% | 2.50% | 0.83% | 0.14% | 3.47% | 2.64% | 2.08% | 0.7960 | 0.1210 |
| *Pyrenula* | 0.00% | 0.00% | 0.06% | 0.02% | 0.03% | 0.00% | 2.84% | 0.96% | 3.23% | 0.94% | 2.34% | 2.17% | 0.4870 | 0.0460 |
| *Geminibasidium* | 0.65% | 0.68% | 1.27% | 0.87% | 0.16% | 4.27% | 0.67% | 1.70% | 0.04% | 0.03% | 1.35% | 0.48% | 0.8270 | 0.5130 |
| *Troposporella* | 0.02% | 0.12% | 0.27% | 0.14% | 0.41% | 0.00% | 3.22% | 1.21% | 3.74% | 0.58% | 0.59% | 1.63% | 0.5130 | 0.0500 |
| *Exophiala* | 2.10% | 1.08% | 0.88% | 1.35% | 2.97% | 0.60% | 0.31% | 1.29% | 0.04% | 0.26% | 0.53% | 0.28% | 0.5130 | 0.0500 |
| *Acrostalagmus* | 0.00% | 0.00% | 0.00% | 0.00% | 0.02% | 0.00% | 3.74% | 1.25% | 0.00% | 0.01% | 4.11% | 1.37% | 0.2460 | 0.2460 |
| *Cylindrocarpon* | 0.97% | 0.30% | 0.02% | 0.43% | 3.25% | 1.72% | 0.38% | 1.78% | 0.15% | 0.21% | 0.46% | 0.27% | 0.1270 | 0.8270 |
| *Tremellodendropsis* | 0.00% | 1.45% | 0.00% | 0.48% | 0.00% | 0.00% | 0.00% | 0.00% | 0.00% | 5.11% | 0.03% | 1.72% | 0.7960 | 0.4870 |
| *Auxarthron* | 0.03% | 0.19% | 0.17% | 0.13% | 0.47% | 5.57% | 0.04% | 2.02% | 0.00% | 0.00% | 0.10% | 0.04% | 0.2750 | 0.1270 |
| *Nectria* | 0.60% | 4.59% | 0.16% | 1.78% | 0.38% | 0.07% | 0.05% | 0.16% | 0.00% | 0.03% | 0.06% | 0.03% | 0.1270 | 0.0500 |
| *Pyrenochaeta* | 1.85% | 2.09% | 0.79% | 1.58% | 1.05% | 0.03% | 0.02% | 0.37% | 0.00% | 0.00% | 0.01% | 0.00% | 0.1270 | 0.0460 |
| *Geoglossum* | 0.00% | 0.01% | 0.58% | 0.20% | 0.00% | 0.14% | 1.70% | 0.61% | 0.04% | 0.00% | 3.37% | 1.13% | 0.6580 | 0.6580 |
| *Phellodon* | 5.26% | 0.00% | 0.50% | 1.92% | 0.00% | 0.00% | 0.02% | 0.01% | 0.00% | 0.00% | 0.05% | 0.02% | 0.2460 | 0.2460 |
| *Trichoderma* | 1.31% | 0.74% | 0.97% | 1.01% | 1.67% | 0.53% | 0.04% | 0.75% | 0.07% | 0.21% | 0.06% | 0.11% | 0.5130 | 0.0500 |
| *Oliveonia* | 0.01% | 0.01% | 5.06% | 1.69% | 0.03% | 0.05% | 0.04% | 0.04% | 0.00% | 0.02% | 0.02% | 0.01% | 0.5130 | 0.8270 |
| *Cladosporium* | 0.24% | 0.34% | 1.00% | 0.53% | 0.13% | 0.42% | 0.03% | 0.19% | 0.02% | 2.38% | 0.09% | 0.83% | 0.2750 | 0.5130 |
| *Acremonium* | 0.27% | 3.75% | 0.15% | 1.39% | 0.34% | 0.07% | 0.00% | 0.14% | 0.00% | 0.00% | 0.00% | 0.00% | 0.2750 | 0.0460 |
| *Hypomyces* | 0.05% | 4.38% | 0.02% | 1.48% | 0.09% | 0.01% | 0.00% | 0.03% | 0.00% | 0.00% | 0.00% | 0.00% | 0.2750 | 0.0370 |
| *Clavicorona* | 0.97% | 0.01% | 2.18% | 1.05% | 0.68% | 0.29% | 0.06% | 0.34% | 0.00% | 0.00% | 0.30% | 0.10% | 0.5130 | 0.1210 |
