# Supplementary material for: Compositional and functional profiling of the rhizosphere microbiomes of the invasive weed Ageratina adenophora and native plants
Source: PeerJ. 2021 Mar 4;9:e10844. doi: 10.7717/peerj.10844 (PMC7937340; doi:10.7717/peerj.10844)
Supplement: Supplemental Information 3 [file peerj-09-10844-s003.docx]

Statistical analyses of the data listed in Table 1

Raw data

| Rhizospheric soil  of plant species | Moisture  ％ | pH | Organic C  (mg Kg^-1^) | NH_4_^+^-N  (mg Kg^-1^) | NO_3_^─^-N  (mg Kg^-1^) | Soluble N  (mg Kg^-1^) | Total N  (%) | Total P  (mg Kg^-1^) | Available P  (mg Kg^-1^) | Total K  (mg Kg^-1^) | Available K  (mg Kg^-1^) |
| --- | --- | --- | --- | --- | --- | --- | --- | --- | --- | --- | --- |
| *Ag. adenophora*  sample 1 | 28.78 | 5.81 | 256.2 | 3.38 | 3.85 | 658.2 | 1.182 | 26.5 | 0.121 | 261 | 0.18 |
| *Ag. adenophora*  sample 2 | 23.7 | 5.18 | 296.4 | 2.18 | 4.74 | 725 | 0.995 | 34 | 0.109 | 302 | 0.2 |
| *Ag. adenophora*  sample 3 | 27.37 | 5.9 | 287.3 | 2.21 | 3.45 | 394.4 | 0.929 | 18.1 | 0.117 | 174 | 0.1 |
| *Ar. indica*  sample 1 | 22.51 | 5.43 | 96.9 | 7.45 | 2.46 | 398.8 | 0.419 | 10.2 | 0.104 | 169 | 0.25 |
| *Ar. indica*  sample 2 | 18.69 | 5.37 | 98.3 | 2.93 | 1.76 | 348.6 | 0.426 | 14.9 | 0.115 | 260 | 0.24 |
| *Ar. indica*  sample 3 | 17.75 | 5.53 | 87.2 | 1.95 | 1.17 | 209.9 | 0.328 | 6.5 | 0.093 | 363 | 0.23 |
| *I. cylindrica*  sample 1 | 25.76 | 5.79 | 41 | 6.33 | 1.07 | 163.4 | 0.173 | 4 | 0.114 | 38 | 0.33 |
| *I. cylindrica*  sample 2 | 26.44 | 6.13 | 74.7 | 2.02 | 1.43 | 72.7 | 0.324 | 12.1 | 0.029 | 155 | 0.44 |
| *I. cylindrica*  sample 3 | 27.51 | 5.81 | 81.9 | 3.37 | 1.15 | 145.2 | 0.299 | 8.5 | 0.11 | 169 | 0.29 |

Statistical analyses (P values) of raw physiochemical data listed in above table. The data from triplicate samples measured for each individual physiochemical parameters of *Ageratina adenophora* rhizosphere soils were compared with either data from rhizosphere soils of native species *Artemisia indica* or *Imperata cylindrica* by using Student T test. Significant differences were established at P < 0.05.

| **Comparison of soil samples** | **Moisture**  **(%)** | **pH** | **Organic C**  **(mg Kg^-1^)** | **NH_4_^+^-N**  **(mg Kg^-1^)** | **NO_3_^─^-N**  **(mg Kg^-1^)** | **Soluble N (mg Kg^-1^)** | **Total N (%)** | **Total P**  **(mg Kg^-1^)** | **Available P (mg Kg^-1^)** | **Total K**  **(mg Kg^-1^)** | **Available K (mg Kg^-1^)** |
| --- | --- | --- | --- | --- | --- | --- | --- | --- | --- | --- | --- |
| *Ag. adenophora*  vs  *Ar. indica* | 0.0295 | 0.4648 | 0.0001 | 0.4315 | 0.0142 | 0.0773 | 0.0014 | 0.0301 | 0.1836 | 0.7995 | 0.0618 |
| *Ag. adenophora*  vs  *I. cylindrica* | 0.9781 | 0.3286 | 0.0003 | 0.3791 | 0.0021 | 0.0113 | 0.0010 | 0.0251 | 0.3245 | 0.0899 | 0.0235 |
